# Anti-VEGF Therapy in Refractory Pituitary Adenomas and Pituitary Carcinomas: A Review

**DOI:** 10.3389/fonc.2021.773905

**Published:** 2021-11-17

**Authors:** Congxin Dai, Siyu Liang, Bowen Sun, Yong Li, Jun Kang

**Affiliations:** ^1^ Department of Neurosurgery, Beijing Tongren Hospital, Capital Medical University, Beijing, China; ^2^ Eight-Year Program of Clinical Medicine, Peking Union Medical College Hospital (PUMCH), Chinese Academe of Medical Sciences & Peking Union Medical College (CAMS & PUMC), Beijing, China

**Keywords:** refractory pituitary adenomas, pituitary carcinomas, VEGF, anti-VEGF, vascular endothelial growth inhibitor

## Abstract

Most pituitary tumors are considered benign adenomas, and only 0.1%–0.2% of them present metastasis and are defined as pituitary carcinomas (PCs). Refractory pituitary adenomas (PAs) lie between benign adenomas and true malignant PCs and are defined as aggressive-invasive PAs, characterized by a high Ki-67 index, rapid growth, frequent recurrence, and resistance to conventional treatments. Refractory PAs and PCs are notoriously difficult to manage because of limited therapeutic options. Vascular endothelial growth factor (VEGF) plays a crucial role in angiogenesis not only during development but also during pathological processes in pituitary tumors. Recently, increasing numbers of preclinical studies and clinical research have demonstrated that anti-VEGF therapy plays an important role in pituitary tumors. The purpose of this review is to report the role of VEGF in the development and pathology of pituitary tumors and the progress of anti-VEGF therapy in pituitary tumors, including refractory PAs and PCs. Previous preclinical studies indicated that cyclin-dependent kinase 5 (CDK5)-mediated VEGF expression might play a crucial role in the development of PAs. Vascular endothelial growth inhibitors have been reported as independent predictors of invasion in human PAs and have been indicated as markers for poor outcome. Furthermore, several studies have reported that angiogenesis decreases tumor sizes in experimental animal models of pituitary tumors. The expression of VEGF is relatively high in PAs; therefore, anti-VEGF therapy has been used in some refractory PAs and PCs. To date, anti-VEGF has been reported as monotherapy, in combination with temozolomide (TMZ), TMZ and radiotherapy, and with pasireotide, which might be a promising alternative therapy for refractory PAs and PCs resistant to conventional treatments. However, the role of anti-VEGF therapy in pituitary tumors is still controversial due to a lack of large-scale clinical trials. In summary, the results from preclinical studies and clinical trials indicated that anti-VEGF therapy monotherapy or in combination with other treatments may be a promising alternative therapy for refractory PAs and PCs resistant to conventional treatments. More preclinical studies and clinical trials are needed to further evaluate the exact efficacy of anti-VEGF in refractory PAs and PCs.

## Introduction

Pituitary adenomas (PAs) are common tumors arising in the anterior pituitary gland with the second highest incidence, representing approximately 10%–15% of intracranial primary tumors (1–3). Most PAs are considered benign tumors that can be cured by surgery and medication. However, a subset of invasive PAs with a high Ki-67, rapid growth, and early recurrences is refractory to conventional treatments such as surgery, medication, and radiotherapy and are referred to as refractory PAs (4). Rarely, 0.1%–0.2% of pituitary tumors can present with either craniospinal dissemination or systemic metastases, which are true malignant tumors and defined as pituitary carcinomas (PCs) (5). Refractory PAs and PCs are notoriously difficult to manage because of limited availability of therapeutic approaches. Recently, temozolomide (TMZ) has been recommended as a first-line treatment for refractory PAs and PCs by the European Society of Endocrinology due to its promising efficacy. However, only approximately 60% of patients show a response to TMZ, and some patients develop resistance during treatment (6, 7). Therefore, the discovery of new therapeutic targets is of particular importance for the management of refractory PAs and PCs. Recent studies have shown that vascular endothelial growth factor (VEGF) and its receptor (VEGFR) play crucial roles in angiogenesis not only in its development but also during pathological processes in pituitary tumors (8). Moreover, an increasing number of clinical case reports have demonstrated that anti-VEGF therapy is beneficial in treating refractory PAs and PCs. Here, this review presents the role of the VEGF/VEGFR pathway in angiogenesis of pituitary tumors and the progress of anti-VEGF therapy in pituitary tumors, including refractory PAs and PCs.

## Angiogenesis in Pituitary Tumors

Angiogenesis, the process of blood vessel growth, is essential for tumor progression and metastasis (9). During angiogenesis, an organized vascular network develops from a primitive vascular network (10). Angiogenesis correlates with the development of metastasis (11–13), recurrence (14), and poor prognosis (15, 16) in many human tumors, including breast, bladder, prostate, and stomach tumors. Contrary to most solid tumors, PA tissue contains fewer blood vessels than normal pituitary glands (17). In particular, not only was the number of vessels much lower but also the size of each vessel was much smaller in PAs than in normal pituitary glands (17–22). The angiogenesis between different PA subtypes is divergent among studies. Jugenburg et al. (22) reported that PAs have significantly lower vascular densities than non-tumorous adenohypophyses. Pituitary prolactin (PRL)-secreting adenomas have the highest vascular densities, and growth hormone (GH)-producing adenomas have the lowest vascular densities. However, no differences were observed between noninvasive and invasive PAs. Primary PCs show no significant increase in vascular densities, but some metastatic tumors exhibit high vascularity. These results indicated that PAs have a limited capacity to induce angiogenesis. Another study demonstrated that the highest counts of immunopositive vascular profiles were noted in follicle-stimulating hormone (FSH)-expressing adenomas, whereas the lowest vascular density was observed in GH-expressing tumors (22, 23). Angiogenesis has been shown to be related to clinical behavior, prognosis, and response to treatment in many different types of PAs. Turner et al. (17, 24) reported that invasive macroprolactinomas were significantly more vascular than noninvasive tumors; however, medical therapy with metyrapone or bromocriptine did not influence angiogenesis in adenomas. Vidal et al. (25) also reported a tendency of invasive PAs to be more highly vascularized than noninvasive PAs; the highest level of microvessel density was found in PCs, while the lowest was found in GH-producing adenomas. Moreover, they demonstrated that the microvessel density of macroadenomas in older patients was significantly higher than that in patients younger than 40 years (25). In summary, PAs are usually less vascularized than normal pituitary glands, while PCs are more vascular than PAs. Although the vascular densities may be related to tumor size, proliferation, hemorrhage, and the treatment response of PAs (19–22, 25), it is still unclear what specific role they play in the tumorigenesis and progression of PAs.

## Vascular Endothelial Growth Factor Expression in Pituitary Tumors

VEGFs are key mediators of endothelial cell proliferation, angiogenesis, and vascular permeability. VEGFs are a family of angiogenic and lymphangiogenic growth factors. VEGF pathways comprise multiple VEGF glycoproteins (VEGFA, VEGFB, VEGFC, VEGFD, and VEGFE) and multiple transmembrane receptors (VEGFR1, VEGFR2, and VEGFR3) (26). VEGFA, commonly referred to as VEGF, has multiple isoforms as a result of alternative exon splicing (27). Although they have various affinities, these isoforms are all capable of binding to VEGFR1 or VEGFR2. VEGFR has intracellular tyrosine kinase activity, which is considered to be the major mediator of the angiogenic properties of VEGF. VEGF binds to the external membrane domain of VEGFR and causes intracellular signaling in endothelial cells, resulting in proliferation and migration (28). VEGF and VEGFR contribute to a potential therapeutic target in a variety of tumors (29–31). VEGF and its receptors are regularly overexpressed in a wide variety of human cancers, including PAs and PCs. Although the concordance of VEGF expression between studies may be poor, in general, VEGF immunoreactivity is moderate to strong in most cases (32). Lloyd et al. (33) analyzed VEGF expression in 148 cases and found positive staining in all subtypes, with a mild to moderate degree in 92.3% (131/142) of PAs and a strong degree in 100% (6/6) of PCs. Fukui et al. (34) also found that VEGF expression was weak in 12.5% (6/48), moderate in 54.2% (26/48), and strong in 33.3% (16/48) in a total of 48 PAs. Wang et al. (35) reported that 58.9% of 197 PAs had strong VEGF expression. VEGF mRNA was detected in more than 85% of PAs and had a significant correlation with VEGF protein expression (32, 36). VEGF expression varies in different subtypes of PAs (33, 35, 37). High VEGF expression was found in nonfunctioning (19, 21, 33, 35, 38) and pituitary adrenocorticotropic hormone (ACTH) (19, 33, 35)-, GH (19, 33, 38)-, PRL (35, 37, 38)-, and FSH (35, 37)-secreting PAs. In tumor tissues, pituitary GH- and PRL-secreting adenomas had diffuse VEGF distribution, while ACTH-, TSH-, and luteinizing hormone (LH)-secreting adenomas showed focal VEGF expression (32, 36, 39). In addition to tumor cells, VEGF mRNA and VEGF expression were mainly present in endothelial cells and folliculostellate cells (36, 40, 41). PCs had significantly higher VEGF mRNA amplification and stronger VEGF immunostaining than those of PAs (33). Therefore, different subtypes of PAs have different levels of VEGF, indicating that anti-VEGF therapy has distinct therapeutic effects on different subtypes of PAs.

VEGF has significant roles in the development of tumor neovascularity and peritumoral edema. Anti-VEGF antibodies removed 75%–99% of the permeability activity (42). Evidence has shown that VEGF is correlated with the pathogenesis of cystic formation in PAs (34). Other features affected by VEGF expression remain controversial. Overexpression of VEGF was associated with intratumoral hemorrhage (43), extrasellar invasion (37, 44), and rapid recurrence (37), although these findings were not significant in other studies (19, 21, 34, 35, 37, 38, 45, 46). Moreover, as shown in several studies, VEGF expression had no relation with tumor size (19, 34, 35, 45) or Ki-67 index (21, 38, 43). Moreover, no clear association was found between microvessel density and VEGF expression (19, 21). The low microvessel density despite VEGF overexpression has caused researchers to ask if inhibitory factors related to VEGF exist in PAs (36). The role of VEGF in the development and progression of PAs is still controversial; however, the expression of VEGF has not yet been used as a conclusive marker of the aggressive behavior of PAs. Current studies indicate that VEGF might play a role in tumoral vascular growth, not by increasing the number of vessels, but by other mechanisms, such as an increase in vascular permeability that favors the abundant diffusion of nutrients.

## Preclinical Studies of Angiogenesis in Pituitary Tumors

Preclinical data indicated that VEGF is a potential therapeutic target in PAs. A previous study demonstrated that VEGF plays a crucial role in tumor angiogenesis during the development of a rat prolactinoma animal model (40). Estrogen-induced prolactinoma expresses a high level of VEGF associated with marked angiogenesis (47). Anti-VEGF resulted in a significant shrinkage in tumor volume, a decrease in the Ki-67 index, and the repair of pituitary vessels (48). Additionally, the characteristic “blood lakes” in prolactinoma were replaced by repaired microvascular structures on three-dimensional (3D) observation under a confocal laser scanning microscope. The current first-line therapy for prolactinomas is dopamine (DA) agonists (Das). Dopamine D2 receptors (D2Rs), which are widely localized in the anterior and intermediate lobes of pituitary glands, can combine with DA to activate signaling cascades (49). DA therapy targeting D2R yields an excellent response in prolactinomas and some clinical benefits in non-prolactinoma pituitary tumors (50). The decrease in D2R expression may explain the resistance to DA. Previous studies have identified the association between VEGF and D2R. In D2R knockout mice, Cristina et al. (51) reported increases in VEGF mRNA transcription, VEGF expression, and highly vascular adenomas. When treating D2R-deficient mice with anti-VEGF, Luque et al. (52, 53) noticed a substantial decrease in serum prolactin, a reduction in tumor size, and a significant decrease in vascularity. Furthermore, anti-VEGF might have additive effects in combination with drugs targeting complementary pathways related to angiogenesis. In mice with hemorrhagic prolactinoma, monotherapy with anti-VEGF or DA can restrain tumor growth and improve vascular remodeling. Only the combination of anti-VEGF and DA can suppress intratumoral hemorrhage (54). In concurrence, prolonged DA treatment enhanced pituitary VEGF expression in wild-type mice (51). These findings provide a provocative possibility of combination therapy with anti-VEGF and DA.

## Therapeutic Targeting of Vascular Endothelial Growth Factors in Pituitary Tumors

### Bevacizumab

**Figure 1:**
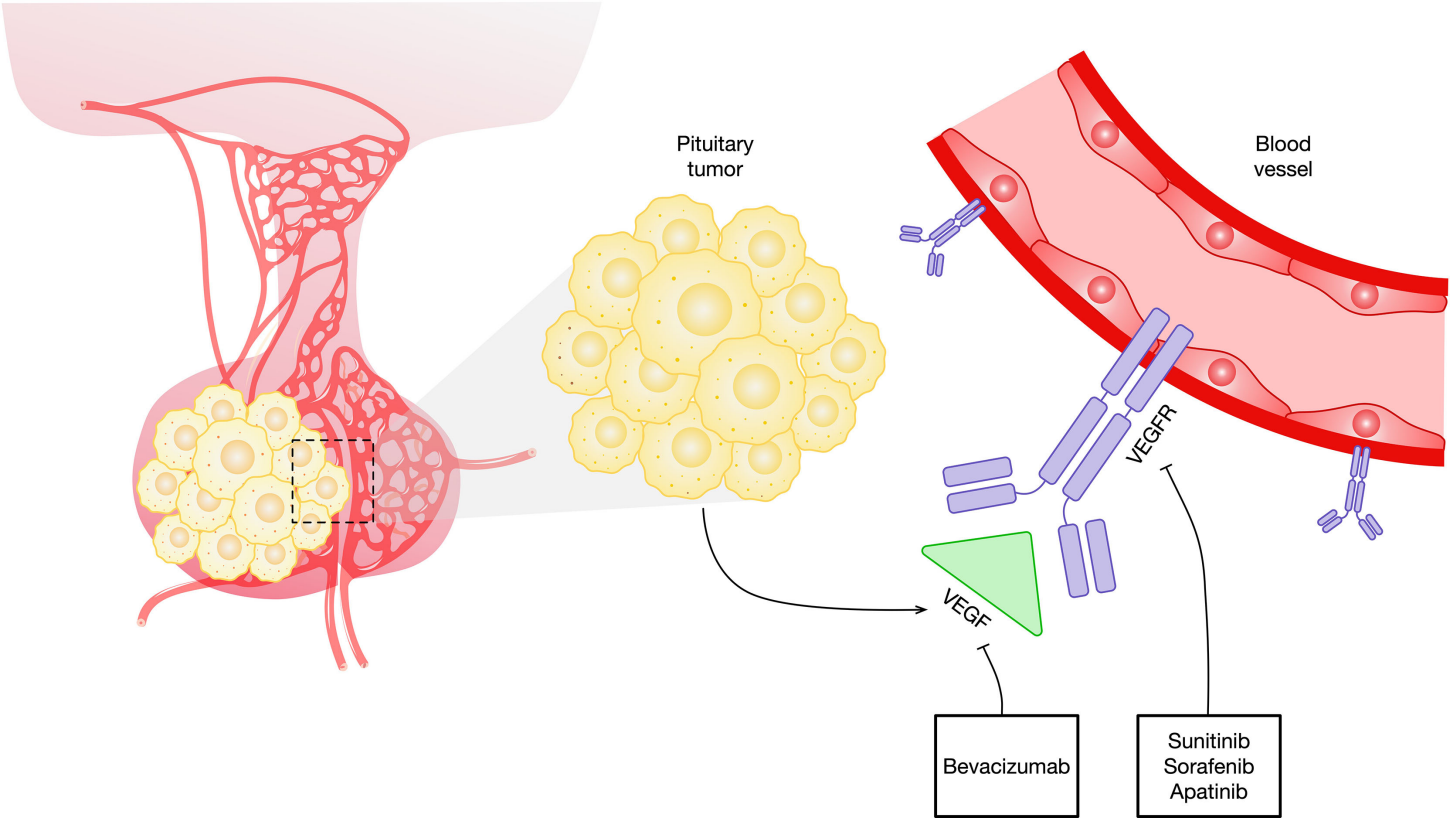
Schematic representation of antiangiogenic agents that target the VEGF and VEGF signaling pathways in pituitary tumors. VEGF, vascular endothelial growth factor; VEGFR, vascular endothelial growth factor receptor.

PAs and PCs highly express VEGF, which is one of the justifications for targeting VEGF and its receptors in this disease. Anti-VEGF has demonstrated significant activity as a single agent in murine studies. The recombinant humanized monoclonal antibody bevacizumab is the first approved agent directed against VEGF (**Figure 1** and **Table 1**). The common side effects of bevacizumab are fatigue, hoarseness, and hypertension. The rare side effects of this agent include clotting, hemorrhage, wound-healing disorders, gastrointestinal perforation, reversible posterior leukoencephalopathy syndrome, and proteinuria (55). Bevacizumab needs to be administered only every 2 or 3 weeks due to its prolonged half-life. This agent can be readily combined with chemotherapy agents, and preclinical evidence indicates synergy for some combinations of chemotherapeutic compounds when used alongside bevacizumab. Bevacizumab has thus far been the drug most tried for targeting the VEGF pathway in pituitary tumors.

**Table 1:** Targets and sites of action of the VEGF angiogenesis receptor and ligand in pituitary tumors.

Target	Agent	Drug class	Site(s) of action
**VEGF**	Bevacizumab	Monoclonal antibody	VEGF
**VEGF receptor**	Sunitinib	TKI	VEGFR1, VEGFR2, PDGFR, KIT, FLT3, and CSF1R
	Sorafenib	TKI	VEGFR2, FLT3, PDGFR, KIT, FLT3, and FGFR1
	Apatinib	TKI	VEGFR2

CSF1R, colony-stimulating factor receptor type 1; FGFR1, fibroblast growth factor receptor 1; FLT3, fms-like tyrosine kinase 3; KIT, stem cell factor receptor; PDGFR, platelet-derived growth factor receptor; TKI, tyrosine kinase inhibitor; VEGF, vascular endothelial growth factor; VEGFR1, vascular endothelial growth factor receptor 1; VEGFR2, vascular endothelial growth factor receptor 2.

The published clinical cases (7, 56–68) are presented in **Table 2**. In this review, we used the same criteria used in the recent European Society of Endocrinology survey (7). A complete radiological response was defined as no visible tumor, partial response (PR) as at least 30% tumor regression, stable disease (SD) as less than 30% regression but no more than a 10% increase, and progressive disease (PD) as more than a 10% increase in tumor size or presentation of new metastasis. For functioning tumors, complete biochemical response was defined as normalization of hormone concentration, PR as more than a 20% reduction in hormone, SD as less than but no more than a 20% change in hormone, and PD as more than a 20% increase in hormone levels. To date, 19 cases treated with bevacizumab have been reported. Among these cases, eight are corticotroph tumors. Three were other subtypes (one somatotroph, one lactotroph, and one null cell), and the subtypes of other cases were not available. The majority of the PAs (8/11) were clinically functioning when the cases were reported; five in 12 cases presented with extracranial metastases, and seven in 12 were diagnosed with PC at the time of data collection. Most of the patients (9/12) underwent more than two surgeries in the sella. All patients received radiotherapy. One hundred percent (10/10) of tumors showed a Ki-67 index ≥10% at the last pathological examination.

**Table 2:** Cases of pituitary carcinomas and aggressive pituitary tumors treated with anti-VEGF.

Ref	ID	Age/Sex	Tumor subtypes	Extent of disease beyond sellar region	Prior surgeries	Prior radiation	Medical treatments	Gene, molecular data	Response to anti-VEGF	PFS after first dose of anti-VEGF (mo)/Outcome
**Anti-VEGF**										
**(** 56 **)**	1	44/M	Corticotroph tumor, nonfunctioning	Intracranial: suprasellar, cavernous sinus, optic chiasm Extracranial: spine	7 CNS surgeries Spine surgery Sellar lesion biopsy	1. Sellar region 2. Vertebral metastases	1. TMZ×8 cycles (PD) 2. TMZ×16 cycles (PD) 3. TMZ×8 cycles (PD) 4. BEV×26 cycles (SD)	VEGF pos MGMT high Pathology after BEV: cell injury, vascular abnormalities, and fibrosis	R: SD	26/Survival
**(** 57 **)**	2	25/F	Corticotroph tumor, functioning	Extracranial: bone	3 CNS surgeries Bilateral adrenalectomy	1. Pituitary fossa	1. SSA (PD) 2. TMZ (PD) 3. BEV and SSA×6 cycles (SD)	NA	R: SD B: PR, plasma ACTH decreased from >200,000 to 113,000 pg/ml	6/Survival
**(** 58, 59 **)**	3	56/F	Corticotroph tumor, functioning	Intracranial: suprasellar, cavernous sinus, optic chiasm, sphenoid sinus	6 CNS surgeries Bilateral adrenalectomy	1. Sellar region	1. SSA×1 mo (PD) 2. CAB×2 mo (PD) 3. TMZ×9 cycles (PD, after withdrawal) 4. BEV (SD)	Ki-67 40% MGMT low	R: SD	NA/Death (postoperative complication)
**(** 60, 61 **)**	4	4/M	Somatotroph tumor, functioning	Intracranial: suprasellar, cavernous sinus, optic chiasm	1 CNS surgeries	1. Sellar region	1. TMZ×3 cycles, TMZ and BEV×35 cycles, PEG (PR, concurrent with surgery and radiotherapy, stopped due to potential gonadal toxicity) 2. PEG and SSA (PR)	Nonsense AIP mutation VEGF pos Ki-67 12% P53 neg MGMT low	R: PR, reduction in pituitary tumor volume B: SD	48/Survival
**(** 62 **)**	5	63/M	Corticotroph tumor, functioning	Intracranial: suprasellar, cavernous sinus, optic chiasm, sphenoid sinus Extracranial: lung	1 CNS surgery	1. Sellar region and/or lung metastasis	1. BEV and TMZ×2 cycles, TMZ×12 cycles (PR, concurrent with surgery and radiotherapy)	Ki-67 50%	R: PR, reduction in lung metastasis volume B: SD	60/Survival
**(** 7 **)**	6	NA	NA	NA	NA	NA	1. TMZ and BEV (PR)	NA	PR	NA
**(** 7 **)**	7	NA	NA	NA	NA	NA	1. TMZ (PD) 2. TMZ and BEV (PR)	NA	PR	NA
**(** 7 **)**	8	NA	NA	NA	NA	NA	1. TMZ (PD) 2. TMZ and BEV (NA)	NA	NA	NA
**(** 7 **)**	9	NA	NA	NA	NA	NA	1. TMZ (PD) 2. TMZ and BEV (NA)	NA	NA	NA
**(** 7 **)**	10	NA	NA	NA	NA	NA	1. TMZ (PD) 2. BEV (SD)	NA	SD	NA
**(** 7 **)**	11	NA	NA	NA	NA	NA	1. TMZ (PD) 2. BEV (PD)	NA	PD	NA
**(** 7 **)**	12	NA	NA	NA	NA	NA	1. TMZ (PD) 2. BEV (NA)	NA	NA	NA
**(** 63 **)**	13	49/F	Corticotroph tumor, functioning	Intracranial: cerebrum Extracranial: bone, liver	3 CNS surgeries Bilateral adrenalectomy	1. Sellar region	1. TMZ (PD) 2. EVE (PD) 3. SUN (PD) 4. BEV (PD)	Ki-67 10%	R: PD B: PD	NA/Death
**(** 64 **)**	14	51/M	Corticotroph tumor, functioning	Intracranial: right temporal lobe, cervico-medullary junction, dural based	2 CNS surgeries	1. Sellar region 2. Cervico-medullary metastasis	1. TMZ×12 cycles and BEV×26 cycles (SD, concurrent with surgery and radiotherapy)	Ki-67 15%	R: SD B: SD	96/Survival
**(** 65 **)**	15	72/F	Lactotroph tumor, nonfunctioning	Intracranial: dura Extracranial: spine	3 CNS surgeries Spine surgery	1. Sellar region 2. Spinal metastasis	1. TMZ×3 cycles (PD) 2. IPI and NIV×2 cycles (SD, stopped due to nephritis) 3. NIV×17 cycles (PD) 4. IPI and NIV×4 cycles (PD, with nephritis and hepatitis) 5. BEV×3 cycles (SD, stopped due to nephritis)	Ki-67 20% MGMT high PD-L1 neg TMB low Mismatch repair deficient neg	R: SD	9/Survival
**(** 66 **)**	16	55/M	NA	Intracranial: suprasellar, cavernous sinus, optic chiasm, left frontotemporal dura, middle cranial fossa	3 CNS surgeries Thyroidectomy	1. Sellar region	1. TMZ×7 cycles (PD, after withdrawal) 2. CCNU×2 cycles (SD, stopped due to poor tolerance) 3. BEV (NA)	Ki-67 13-25.5% P53 neg MGMT low	NA	NA/Death
**(** 67 **)**	17	NA	Corticotroph tumor, functioning	Intracranial: cavernous sinus	5 CNS surgeries Bilateral adrenalectomy	1. Sellar region	1. SSA (SD, stopped due to poor tolerance) 2. CAB (PD) 3. TMZ×7 cycles (PD, after withdrawal) 4. BEV×2 cycles (PD)	VEGFR pos Ki-67 8-20% P53 pos	R: PD B: PD	1/Death
**(** 67 **)**	18	NA	Corticotroph tumor, functioning	Intracranial: cavernous sinus, clivus	5 CNS surgeries Bilateral adrenalectomy	1. Sellar region	1. SSA (PD) 2. TMZ×3 cycles (PD) 3. CAB and TMZ×3 cycles (PD) 4. BEV×1 cycles (PD)	VEGFR pos Ki-67 5-10% P53 pos	R: PD B: PD	1/Death
**(** 67 **)**	19	NA	Null cell tumor	Intracranial: cavernous sinus	5 CNS surgeries	1. Sellar region	1. TMZ×6 cycles (PD) 2. BEV×6 cycles (SD)	VEGFR pos Ki-67 5-10% P53 neg	R: SD B: SD	18/Death (postoperative complication)
**Anti-VEGFR**										
**(** 7 **)**	20	NA	NA	NA	NA	NA	1. TMZ (PD) 2. SUN (PD)	NA	PD	NA
**(** 68 **)**	21	41/F	Somatotroph tumor, functioning	Intracranial: suprasellar, cavernous sinus, optic chiasm, clivus	4 CNS surgeries	1. Sellar region	1. SSA×2 mo (PD) 2. TMZ and APA×12 cycles (SD, concurrent with surgery)	VEGFR pos Ki-67 5-10%	R: SD B: PR, plasma GH decreased from 10 to 1.5 ng/ml	31.5/Survival

AIP, aryl hydrocarbon receptor-interacting protein; APA, apatinib; B, biochemical criteria; BEV, bevacizumab; CAB, cabergoline; CCNU, 1-(2-chlorethyl)-3-cyclohexyl-1-nitrosurea; CNS, central nervous system; EVE, everolimus; F, female; IPI, ipilimumab; IHC, immunohistochemistry; M, male; MGMT, O^6^-methylguanine-DNA methyltransferase; mo, months; NA, not available; neg, negative; NIV, nivolumab; PD, progressive disease; PD-L1, programmed death-ligand 1; PEG, pegvisomant; PFS, progression-free survival; pos, positive; PR, partial response; R, radiological criteria; Ref, reference; SD, stable disease; SSA, somatostatin analogs; SUN, sunitinib; TMB, tumor mutational burden; TMZ, temozolomide; VEGF, vascular endothelial growth factor; VEGFR, vascular endothelial growth factor receptor.

Of the 12 patients to whom TMZ was administered prior to bevacizumab, all yielded PD. A second course of TMZ was administered to two patients (one on monotherapy, one on TMZ combined with cabergoline), which resulted in further progress. Notably, O^6^-methylguanine-DNA methyltransferase (MGMT) immunohistochemistry was observed to be low in two and high in two. None of the four cases responded to TMZ. Bevacizumab was chosen as the second- or third-line therapy after TMZ failed. Six patients achieved SD [five on monotherapy, one on somatostatin analog (SSA) + bevacizumab], and four had disease progression. Ortiz et al. (56) reported an aggressive silent corticotroph cell PA that progressed to carcinoma despite TMZ administration and was subsequently treated with bevacizumab, achieving 26 months of SD, as documented on serial MRI and positron emission tomography scans. Bevacizumab therapy resulted in severe cell injury, vascular abnormalities, and fibrosis in tumors. This case first revealed the effectiveness of targeting VEGF in blocking angiogenesis, thus inhibiting tumor growth. VEGF immunoreactivity was positive in this case. However, VEGF/VEGFR immunoreactivity may not directly demonstrate efficacy. In another three patients with VEGFR expression in PC, two showed poor responses to bevacizumab (67).

In the other seven cases, bevacizumab was administered in parallel with TMZ as the first-line therapy. Although the outcomes were not available in two cases, PR or SD was reported in five patients, including one who failed to receive TMZ as a first-line therapy. Preclinical studies showed that most PAs exhibited low expression of MGMT and high expression of VEGF, while the expression of VEGF was positively associated with MGMT (35). TMZ and bevacizumab might be considered a combination therapy under the premise of indications. Touma et al. (62) reported a patient with ACTH-secreting PC who received adenomectomy in combination with radiation, TMZ, and bevacizumab and was kept in remission over 5 years of follow-up after therapy. Rotman et al. (64) reported a comparable result in another case with a corticotroph PC. The patient underwent surgery and radiotherapy for metastasis, followed by combined, overlapping chemotherapy with TMZ and bevacizumab, leading to a progression-free survival of 8 years. In the ESE survey (7) on 166 patients with aggressive PAs or PCs, seven were administered bevacizumab once, as shown in **Table 2**. Three patients were treated with bevacizumab monotherapy, resulting in SD in one patient and PD in one patient. Four patients took bevacizumab combined with TMZ, and 50% (2/2) had PR. These observations are consistent with other studies that have shown complementary effects of anti-VEGF combined with drugs targeting alternative pathways implicated in angiogenesis and further underline the importance of combination therapies when choosing bevacizumab.

Importantly, bevacizumab is a new option in the treatment of aryl hydrocarbon receptor-interacting protein (AIP)-related PA. Inactivating germline mutations in the AIP gene are linked to PA predisposition. Korbonits et al. (60) and Dutta et al. (61) treated a 4-year-old child diagnosed with AIP-mutated somatotroph PA with combination therapy of TMZ and bevacizumab concomitantly with radiation and pegvisomant, which stabilized tumor growth and hormone secretion over 4 years. This case revealed that bevacizumab could play a role in controlling genetically driven refractory PAs.

### Tyrosine Kinase Inhibitors

Although bevacizumab has been the most studied VEGF inhibitor in pituitary tumors, various other agents are in development (**Table 1**). The majority of these agents are tyrosine kinase (TK) inhibitors. Sunitinib and sorafenib are small molecules that inhibit multiple TK receptors, some of which are implicated in angiogenesis, tumor growth, and metastatic progression (**Figure 1**) (69–71). Sunitinib and sorafenib have been approved in different clinical scenarios such as advanced renal cell carcinoma (72) and local or metastatic thyroid carcinoma refractory to radioactive iodine treatment (73) and hence are used in the treatment of pituitary metastasis from renal cell carcinoma (74–81) and thyroid carcinoma (82, 83). Apatinib, also known as rivoceranib, is a TK inhibitor that selectively targets VEGFR (**Figure 1**) (84). The toxicity and side-effect profile of TK inhibitors varies as a function of their target TKs, including hematological events (anemia, neutropenia, and thrombocytopenia), diarrhea, nausea, fatigue, hypertension, skin rash, elevation of liver enzymes, and proteinuria.

Sunitinib has been reported in the treatment of PAs and PC in two cases thus far. Both cases had observed PD (**Table 2**). Apatinib was administered in a 41-year-old female in combination with TMZ as a second-line treatment (68). This patient was diagnosed with GH-secreting recurrent PA that resisted surgeries, radiation, and SSA. As VEGFR was expressed in the tumor, apatinib and TMZ were recommended. She achieved stabilization in the tumor and a decrease in serum GH levels over a period of 31.5 months of follow-up.

TK inhibitors might represent a therapeutic target in PAs associated with somatic genetic defects. Multiple endocrine neoplasia type 1 (MEN1) is an autosomal dominant disorder characterized by tumors of the pituitary gland, parathyroid gland, endocrine-gastrointestinal tract, and pancreas. In patients with MEN1, PAs are usually diagnosed at an earlier age, have higher degrees of aggressiveness and invasiveness, are more often resistant to treatment, and have higher risks of recurrence than sporadic PAs (85). Murine studies support that targeted angiogenesis in MEN1 leads to an obvious inhibition of pituitary tumor growth and hormone secretion and a significantly increased tumor-free survival time. Additionally, the vascular density in pancreatic islet tumors was significantly reduced by the treatment (86). Sunitinib was approved to treat locally advanced or metastatic pancreatic neuroendocrine tumors and refractory gastrointestinal stromal tumors (87, 88). Sunitinib has also been studied in MEN1 syndrome (89–92). However, data are still limited to drive any conclusion on the treatment of MEN1-related PAs.

To date, although attempts at bevacizumab and TK inhibitors in pituitary tumors have not gone beyond case studies, the anti-VEGF/VEGFR pathway has shown promise as an alternative therapy for patients with refractory PAs and PCs resistant to conventional treatments. Furthermore, the anti-VEGF/VEGFR pathway in combination with TMZ, TMZ and/or radiotherapy with SSA might have a synergistic therapeutic effect. However, the specific efficacy of the anti-VEGF/VEGFR pathway in patients with refractory PAs and PCs still needs further large-scale prospective clinical trials for confirmation.

## Conclusion

In summary, the results from preclinical studies and clinical trials indicated that anti-VEGF monotherapy or in combination with other treatments may be promising alternative therapies for patients with refractory PAs and PCs resistant to conventional treatments. However, more preclinical studies and large-scale prospective clinical trials are needed to further evaluate the exact efficacy of anti-VEGF in pituitary tumors.

## Author Contributions

All authors listed have made substantial, direct, and intellectual contribution to the work and approved it for publication.

## Funding

Financial support for this study was provided by the Scientific Research Project of Capital Health Development in 2018 (grant number: 2018-4-4018), the CAMS Innovation fund for Medical Science (grant number: CIFMS, 2017-12M-2-005), and the Beijing Natural Science Foundation (grant number: 7182137). The funding institutions had no role in the design of the study, data collection and analysis, the decision to publish, or the preparation of the article.

## Conflict of Interest

The authors declare that the research was conducted in the absence of any commercial or financial relationships that could be construed as a potential conflict of interest.

## Publisher’s Note

All claims expressed in this article are solely those of the authors and do not necessarily represent those of their affiliated organizations, or those of the publisher, the editors and the reviewers. Any product that may be evaluated in this article, or claim that may be made by its manufacturer, is not guaranteed or endorsed by the publisher.
